# Reduced dosing and liability in methadone maintenance treatment by targeting oestrogen signal for morphine addiction

**DOI:** 10.1111/jcmm.13266

**Published:** 2017-07-12

**Authors:** Yao‐Chang Chiang, Ruey‐Yun Wang, Chieh‐Liang Huang, Shue‐Hwa Chen, Wen‐Jing Ho, Hsien‐Yuan Lane, Ing‐Kang Ho, Hwei‐Ting Yang, Wen‐Lung Ma

**Affiliations:** ^1^ Center for Drug Abuse and Addiction Brain Disease Research Center China Medical University Taichung Taiwan; ^2^ Department of Nursing Division of Basic Medical Sciences Chang Gong University of Science and Technology Chiayi County Taiwan; ^3^ Department of Public Health China Medical University Taichung Taiwan; ^4^ Department of Psychiatry China Medical University Hospital Taichung Taiwan; ^5^ Sex Hormone Research Center China Medical University Hospital Taichung Taiwan; ^6^ Department of Nutrition China Medical University Taichung Taiwan; ^7^ Graduate Institution of Clinical Medical Science China Medical University Taichung Taiwan; ^8^ Department of Nursing Asia University Taichung Taiwan

**Keywords:** methadone maintenance treatment, opiate addiction, estrogen, tamoxifen

## Abstract

Methadone maintenance treatment (MMT) is the major tapering therapy for morphine addictive patients. There have gender differences reported in response to MMT. This study discovered that the estrogen‐response element single nucleotide polymorphism (ERE‐SNP; rs16974799, C/T) of cytochrome 2B6 gene (*cyp2b6*; methadone catabolic enzyme) responded differently to MMT dosing. Oestradiol was associated with high MMT dosing, high enantiomer (R‐ or S‐) of 2‐ethylidene‐1,5‐dimethyl‐3,3‐dipheny‐pyrrolidine (EDDP; methadone metabolite) to methadone ratio and increased drug‐seeking behaviour, implicating oestradiol‐CYP‐EDDP/methadone axis decreasing MMT efficacy. In mouse model, oestrogen mitigates methadone antinociceptive response, facilitates methadone catabolism and up‐regulates methadone‐associated metabolizing enzymes. Oestrogen also ablates chronic methadone administration‐induced rewarding response. Mechanism dissection revealed the CC genotype of CYP2B6‐ERE‐SNP exerts higher ERE sequence alignment score, higher estrogenic response as compared to TT genotype. At last, preclinical study *via* targeting estrogen signal that tamoxifen (TMX; selective estrogen receptor modulator, SERM) could facilitate the tolerance phase rewarding response of methadone. Strikingly, TMX also reduces tapering/abstinence phases methadone liability in mice. In conclusion, this study demonstrates altering methadone metabolism through targeting estrogen signals might be able to free morphine addictive patients from the addiction of opioid replacement therapy. Therefore, the add‐on therapy clinical trial introducing SERM in MMT regimen is suggested.

## Introduction

Opioid drugs are the most effective therapeutic analgesic for chronic and cancer pain 1. Continual use of opioids, however, results in the development of tolerance and dependence 2, 3. Moreover, abuse of opioids (heroin and/or morphine) is a serious social problem worldwide (SAMHSA, 2014; http://www.samhsa.gov/prevention). Methadone, a synthetic mu‐ (μ‐)opioid receptor (MOR) agonist, was developed and first introduced to replace morphine for pain treatment 4. It is also considered as most effective alleviating agent for heroin‐addiction withdrawal syndrome 5, 6, 7. One major purpose of introducing opioid replacement therapy is to provide opportunity for social supporting systems to help patients get rid of opioids agents. Although methadone has been shown to be effective in reducing withdrawal symptoms and impulsive injection of opioids 8, chronic use of methadone has also exhibited addictive liability and respiratory repression in some patients 9.

Methadone is major metabolized in the liver through CYP3A4, CYP2D6, CYP2B6, CYP2C19 and CYP1A2 (cytochrome p450s; xenobiotics oxidizing enzyme) through which converting methadone to inactive metabolites (EDDP; 2‐ethylidene‐1,5‐dimethyl‐3,3‐diphenylpyrrolidine) 10, 11. Oral taken methadone subjected to first‐pass effect and could be detected in the plasma after 30 min. of administration. And the methadone bioavailability ranges widely from 41–76% to 85–95% in different patients 10. The discrepancies of bioavailability in patients reflect on pharmacokinetic data that a noticeable variation of plasma EDDP/methadone concentration observed in same dosing patients 12. CYP enzymes modulator has been demonstrated to alter plasma concentration of methadone 10. For example, the off target effect of efavirenz could increase CYP3A4 activity, decrease 60% plasma concentration of methadone; therefore, an increase of 66–133% or 15 mg more methadone dosing is required to alleviate withdrawal symptoms 13, 14. It implicates a therapeutic benefit of CYP suppressor in MMT regimen.

Gender in substances‐abuse individuals has been reported in terms of severity, craving, medical conditions and impairment in associated central nervous system (CNS) functional areas 15, 16, 17, 18. Except for neuronal activity, it has been reported that sex hormones regulate differential panels of liver CYPs, namely feminine or masculine CYPs 19, 20, 21, through which explained the mechanisms of sexual dimorphism on drugs and xenobiotics. Oestrogen (17β‐estradiol, E2) is the active ligand for Estrogen receptors (including ERα and ERβ) which expressions in liver throughout life 22, 23. Genome‐wide association studies reported that the SNPs related to CYPs, opioid receptors, sex hormone producing enzymes provides possible explanation to the individualized methadone dosing in clinics 24. Methadone metabolism is linked to MMT efficacy and also associated to genders. Different metabolic enzymes are involved in enantiomeric S‐ and R‐methadone metabolism 10. S‐methadone can be catabolized to S‐EDDP (2‐ethylidene‐1,5‐dimethyl‐3,3‐diphenylpyrrolidine) by CYP2B6 25, where R‐methadone can be catabolized to R‐EDDP by CYP2C19 26. Luk *et al*., (2010) reported that methadone suppressed oestrogen levels through CYP2C19 expressions 27. In addition, the methadone clearance was increased during pregnancy, which may be due to the raising of CYP2B6 activity and mRNA by increased oestrogen levels 28. These results indicate that the activity of CYP enzymes, including CYP2C19 and CYP2B6, may be regulated by sex hormones to influence methadone effectiveness. Moreover, the gender effects were also exhibited in choices of abuse drugs and addictive behaviours. A study of MMT patients in a single cohort study suggested that males tend to use as their opiate of choice and are more likely to combine with cannabis, while females are more likely to select the street methadone with ketamine, benzodiazepines and/or amphetamines 29. In addition, there is a tendency of abusing opioids shift from prescription analgesics to street methadone for solving the analgesics liability in women 29. This indicates sex hormones signals might be involved in regulating the emotion and motivation for opioids preference.

This study intended to decrease MMT dosing and hopefully to reduce the opioid liability, from female hormone perspective. In the current report, we have dissected oestrogen signals in genetics, serology/hormonal to the regulations of methadone metabolomics and have conducted a preclinical trial that target oestrogen signals by SERM in MMT program.

## Materials and methods

### Study subjects

All the human studies of experimental procedures were approved in accordance with the China Medical University Hospital (CMUH) and Changhua Christian Hospital (CCH) Institutional review board (DMR94‐IRB‐007), and each subject informed consents were documented. We recruited two cohorts (Table S1); all patients were diagnosed by experienced research psychiatrists in accordance with the fourth edition of Diagnostic and Statistical Manual of Mental Disorders (DSM‐IV) guideline (http://www.dsm5.org/Pages/Default.aspx). The cohort 1 heroin abusers were recruited from a hospital‐based study, the cohort 1 was to develop the hypothesis that whether oestradiol modulates the methadone dosing. The cohort 1 was consisted of 326 heroin abusers (age range of 20–70 years) recruited from psychiatric outpatients in CMUH from 2010 Jan. to 2013 Dec. in Taichung, Taiwan. A Total of 90 patients in cohort 2 were collected from CCH (25 patients) and CMUH as a validation cohort from 2013 Jan. to 2014 Dec. In addition, each patient receiving methadone therapy for at least 6 months and keeping unchanged dose for at least 4 weeks before recruitment. Patients who receive medicines currently, may affect methadone metabolism, or any accompany with DSM‐IV Axis I or II psychiatric disorders were excluded in this study. At the time of enrolment, two cohorts of all patients were requested to complete the constructed questionnaire, including demographic data and heroin addictive behaviour survey.

### HUC questionnaire

This study designed a 14‐item questionnaires, heroin using and craving (HUC) to evaluate the behaviours, for heroin abusers received methadone maintenance therapy 30. Each question scores 0–4. The questionnaire is divided into two parts: part I (HUC 1–HUC 6) is to assess the urge for heroin and whether one can shift the attention from heroin. The part I sum score is calculated range from 0 to 24. The part II (HUC 7–HUC 14) is to investigate the daily or weekly frequencies of heroin usage, daily life disturbance, anxiety emotion and the ability to overcome heroin use. The part II sum calculated score ranges from 0 to 32. The higher the score, the more severe the cravings for heroin use.

### **Animals housing**

All male and female C57BL/6 mice (From National Laboratory animal centre, Taiwan) were reared at the CMU (China Medical University) Animal Center, followed the regulation of Taiwan MOST and NIH animal protocol, and approved by Laboratory Animal Welfare Committee (CMU protocol #103‐30‐N). All mice acclimatized to a room with controlled temperature (25°C), humidity (50 ± 10%) and a 12‐hr day–night cycle (light on 08:00–20:00 hrs) for at least 7 days before experimentation. Animals were housed 3–5 per cage and provided with food (Prolab 2500 Rodent 5P14, LabDiet, PMI Nutrition International, St Louis, MO, USA) and water *ad libitum*. The mouse age 6–12 weeks and weighing 20–30 g was used for the experiments. Animals were handled for 3–4 days before the experiments performed. On the test day, animals were transported to the testing room and to habituate with the environment at least for 1 hr. All the animal experiments were performed in accordance with the ethical guidelines which was approved by CMU Animal Core and Use Committee (CMU animal protocol: 103‐30‐N) and followed throughout the study.

### Ovariectomy (OVX) surgery

The procedure of OVX on female mice follows previous report 31. In brief, 10–14‐weeks of female mice were anaesthetized and lower flanks open wound (0.3~0.5 cm) were created, the ovaries were removed, the wound was cleaned and closed by #0.5‐suture lines. After 2‐week recovery, the mice were subjected to experiments.

### E2/ TMX injection Protocol

The E2/TMX injection procedure followed previous work 32. In brief, 17β‐oestradiol (Sigma‐Aldrich, Carlsbad, CA, USA) or (TMX; Sigma‐Aldrich) was dissolved in sesame oil:ethanol (9:1, v:v) [vehicle]. Each mouse was injected subcutaneously (s.c.) with either 0.01 ml vehicle or vehicle containing 20 μg E2 or 100 μg TMX for consecutive 4 weeks, 3 times/week.

### Tail flick assay

The tail flick test was carried out on mice using a modified method of Dai *et al*. 33. The tail flick latency was defined by the time (sec.) the animal withdrew the tail from a heat source (bulb, 8 V/50 W, OSRAM, Germany) and was measured using a semi‐automated machine (Columbus Instruments, Columbus, OH, USA). The infrared intensity of the tail‐flick machine was set at eight, which produced a baseline tail flick latency of 2–3 sec. and the cut‐off time was set as 10 sec. to prevent tissue damage. The rat was put in a restrainer for 5 min. for adaptation before the tail‐flick test was performed. To measure the analgesic effect of opioid agonists, animals were subjected to the tail‐flick procedure once a day to minimize the learning effects. All experimental animals were randomly assigned from different cages to ensure a general effect in the population. The antinociceptive effects were presented as the area under the time–response curve (AUC = latency × time).

### Conditioned place preference (CPP) test

The CPP apparatus used is a two‐compartment acrylic plastic box. The box has two equal‐size compartments (20 × 20 × 25 cm) with a connection 10 × 10 cm door, one with black on the four walls and floor as a visual cue, and the other all in white. Patients were randomly placed into the apparatus and given free access to the entire box (door open) for 15 min. to measure the pre‐drug preference. During the conditioning, the connection door was closed. Methadone (initiation dose was 10 mg/kg for 14 days then decreased half every 5 days) w/o (TMX; 100 μg/mice/day) was paired with the non‐preferred white compartment, while the vehicle was paired with the black compartment. Animals were kept for 1 hr in the corresponding compartment with the connection doors closed. The paired condition was performed one white and one black a day with 6‐hr interval. The days of post‐drug place preference examination were shown in Figure 3A. The post‐drug place preference was conducted for 15 min. Behaviours of the animal were recorded by video tracer software (Trace Mouse II, SINGA, Taipei, Taiwan).

### Tissue preservation and quantitative real‐time Reverse‐transcription polymerase chain reaction (qRT‐PCR)

Mice were sacrificed following the treatment and behavioural testing schedule of methadone. Immediately, the liver was dissected and frozen in liquid nitrogen. Total RNA was isolated from the tissue using the Trizol™ reagent (Invitrogen, Waltham, Massachusetts, USA) according to the manufacturer's protocol. The mRNA level of CYP enzymes will be measured by qPCR using the Bio‐Rad CFX 96 sequence detection instrument. The level of CYP enzymes mRNA was normalized with GAPDH mRNA. The SYBR probe (Bio‐Rad, Hercules, CA, USA) was used as the fluorogenic probe to determine the threshold cycle (Ct), and the forward and reverse primers are shown in Table S2A.

### SNP variants selection

All SNPs of methadone metabolism‐related enzymes (CYP1A2, CYP2B6, CYP2C19 and CYP2D6) were analysed (from UCSC genome browser; http://genome.ucsc.edu). The putative estrogen response element (ERE) area were allocates by comparison the results predicting by TFSEARCH website (http://www.cbrc.jp/research/db/TFSEARCH.html) and PReMod 34 database (http://genomequebec.mcgill.ca/PReMod/). Then, the genotypes of ERE‐SNP alignment score were performed by The BEST (The Binding Element Searching Tool; http://thebest.binfo.ncku.edu.tw/thebest/) algorithm.

### DNA isolation and genotyping

Genomic DNA was extracted from 8‐ to 10‐ml peripheral whole blood samples using the MasterPure™ DNA Purification Kit for Blood Version II (Epicentre, Madison, WI, USA). DNA specimen was dissolved in TE buffer and stored at −20°C until PCR. All the ERE‐SNPs (Table S1) were carried out using Sequenom iPLEX MALDI‐TOF, matrix‐assisted laser desorption ionization time of flight (Sequenom Inc., San Diego, CA, USA), according to the manufacturer's protocol. The primers for each ERE‐SNPs site used are shown in Table S2B.

### ERE‐SNP reporter plasmid construction

CYP2B6‐ERE‐SNP pGL3‐luciferase construction: The patient DNAs of various genotypes were subjected to PCR (polymerase chain reaction) using the following primers (with XhoI and HindIII restriction sites). Primers for CYP2B6, rs16974799 (Intron 1) C/T‐genotype, amplicon = 87‐bp: Forward primer (Xho I): 5′‐CGCTCGAGGCAAGACCCTGTCTCTT‐3′; and Reverse primer (HindIII): 5′‐GCAAGCTTTAACAGAGGCATGATTTGTGC‐3′. And primers for CYP2B6, rs3760657 (5′‐promoter) A/G‐genotype, amplicon = 61‐bp: Forward primer (Xho I): 5′‐CGCTCGAGTCGGTGCTTCACCCTGG‐3′; Reverse primer (HindIII): 5′‐GCAAGCTTCCCAGGAGGAGCAGACA‐3′ The CYP2B6‐ERE‐SNP‐Luciferase plasmids sequences were confirmed with sequencing result (data not show).

### Chemicals, reagents and cell culture

Methadone (USP, USA) was dissolved in distilled water and was administered subcutaneously (*s.c*.) in a volume of 1.0 ml/kg bodyweight: oestradiol (E2; Sigma‐Aldrich); TMX (Sigma‐Aldrich). The human hepatoma cells (HepG2; ATCC, US) were maintained in DMEM medium with 10% FCS (Invitrogen), 1% L‐Glutamine and 1% penicillin/streptomycin as described previously 35.

### Transfection and dual luciferase assay

The assay was performed as previously described 36, 37. Briefly, CYP2B6‐ERE‐SNP‐luciferase and pRL‐TK (thymidine kinase promoter‐driven renilla luciferase plasmid) were co‐transfected into cells using liposomes 38. Cells were then treated, lysed (Promega, WI, USA) and subjected to a Dual‐luminescence reader (Promega).

### Enantiomeric EDDP/methadone detection by LC‐MS/MS

The sample preparation and measurement of R/S‐form enantiomeric methadone or EDDP were modified form previous articles 39, 40. In brief, sample was prepared as follows: standard (R, S) methadone or EDDP was purchased from Sigma (Sigma‐Aldrich). For (R,S) methadone and EDDP, the calibration curve points were: 0, 100, 250, 500, 1000 and 2000 ng/ml. LC/MS/MS analysis was performed on an API 2000 LC/MS/MS system (AB Sciex, Ontario, Concord, Canada). It was interfaced to a HPLC pump equipped with an autosampler (1100 series, Agilent, Waldbronn, Germany). Fifty microlitres of plasma samples and 100 μl of internal standard (EDDP‐D3) were mixed and filtered for use. After 2 min. of vortex, the sample was centrifuged under 15000× *g* for 15 min. and the supernatant was applied for LC/MS/MS analysis. A Chiralcel OD‐R column (250 × 4.6 mm, 5 μm particle size, Daicel Chemical Industries Ltd., Tokyo, Japan) was used, and the isocratic mobile phase system was performed under 0.5 ml/min. of flow rate (phase A: 0.1% formic acid in acetonitrile, phase B: 10 mM ammonium acetate). Q1/Q3 of EDDP was 278.3/234.3, methadone was 310.1/265.6 and EDDP‐D9 was 319.3/268.3.

### E2 detection by ECLIA assay

Electro Chemi Luminescent immunoassay (ECLIA) was used for the quantitative determination of oestrogen in mouse serum on a Roche Elecsys 2010 instrument (Roche, Basel, Switzerland) according to the manufacturer instructions. The chemiluminescence reaction for the detection of the reaction complex is initiated by applying a voltage to the sample solution resulting in a precisely controlled reaction. Serum oestradiol values are given as picograms per millilitre (pg/ml) (pg/ml × 3.67 =  pmol/l). The functional sensitivity of the oestradiol assay was 5 pg/ml (18.4 pmol/l) with a total analytical sensitivity of <5%.

### Statistical analysis

Hardy–Weinberg equilibrium was tested by chi‐square test for goodness of fit. Student's t‐test was used to assess the difference between luciferase activity and genotypes. In addition, we also employed Student's t‐test to compare the different methadone doses, sex hormone in gender during trials. Furthermore, the correlations between sex hormone, behaviour score and EDDP/methadone ratios were analysed. The significance level was set at a two‐sided *P* < 0.05. All statistical analyses were carried out using sas version 9.4 (SAS Institute, Inc, Cary, NC, USA).

## Results

### Oestrogen signal increases MMT dosing

As described, sexes and related hormones could contribute to opioid actions, including MMT dosing in patients. In order to examine the association of oestrogen‐CYP‐MMT dosing in human beings, we recruited first cohort‐based study to associate oestrogen‐related CYPs gene polymorphism with maximal methadone dose (Max) in MMT patients. We have evaluated the association of 10 putative ERE‐SNP sites and Max MMT in the cohort (Table S3). In Table 1, we found the Max MMT dosing in ERE‐SNP (rs16974799; C:T); *CYP2B6* is significantly higher (*P* = 0.0441) in CC genotype compared to TT and CT genotypes. We then performed association of ERE‐SNPs genotypes with Max MMT dosing and found dominant model (*P* = 0.0147) of rs16974799 in patients. Patients carried TT genotype (48.2%) taking lower dose among three MMT dosing groups (Table 1). Next, we conducted second cohort study to observe the association of sexes and female hormones with MMT dosing (pMax: maximal MMT dosing before enter the trial; SS: steady‐state MMT dosing in the trial; Max: maximal MMT dosing during the trial). In our validation cohort study (Table 2), we found either pMax, SS and Max MMT dosing are gender‐related. Females were significantly correlated with higher MMT dosing compared to males. While associate female hormones (oestradiol, E2 and progesterone, P4) to MMT dosing, it's E2 (*P* = 0.0472) but not P4 (*P* = 0.4576) is associated with Max MMT in the trial. This result implicating oestrogen is the confounder in the sexual dimorphic MMT dosing.

**Table 1:** ERE‐SNP (CYP2B6 rs16974799) Genotypes association to MMT dosing of cohort 1

Genotype		≤50 mg (*n* = 79)	51~99 mg (*n* = 127)	≥100 mg (*n* = 64)	*P*‐value
	CC	41 (51.9%)	83 (65.4%)	48 (75.0%)	0.0441*
	CT	33 (41.8%)	41 (32.3%)	15 (23.4%)	
	TT	5 (6.3%)	3 (2.4%)	1 (1.6%)	
Dominant	CC+CT	41 (51.9%)	83 (65.4%)	48 (75.0%)	0.0148*
	TT	38 (48.2%)	44 (34.7%)	16 (25.0%)	
Recessive	CT+TT	74 (93.7%)	124 (97.6%)	63 (98.4%)	0.2024
	CC	5 (6.3%)	3 (2.4%)	1 (1.6%)	

pMax, Maximal MMT dose before enter this trial; SS, steady‐state MMT dose in this trial; Max, Maximal MMT dose during this trial; avg, average; S.D., standard deviation; [E2], 17β‐oestradiol; [P4], pregn‐4‐ene‐3,20‐dione (progesterone). *P < 0.05; **P < 0.01.

**Table 2:** Variables association to MMT dosing in cohort 2

	Gender (*n* = 90)		*P*‐value	[E2] (pg/ml; *n* = 89)		*P*‐value	[P4] (pg/ml; *n* = 87)		*P*‐value
	Male (*n* = 66)	Female (*n* = 24)		≤20 (*n* = 45)	>20 (*n* = 44)		<0.4 (*n* = 45)	≥0.4 (*n* = 42)	
pMax (avg.( S.D.))	80.98 (31.89)	112.71 (57.54)	0.016*	82.67 (43.15)	96.36 (41.45)	0.1305	86.56 (39.57)	92.62 (46.71)	0.5144
SS (avg.( S.D.))	48.98 (34.46)	90.39 (60.93)	0.0038**	54.14 (47.33)	66.03 (46.01)	0.2329	62.04 (43.99)	58.89 (50.17)	0.7556
Max (avg.(S.D.))	82.20 (31.78)	122.50 (61.49)	0.0048**	83.56 (43.09)	102.6 (46.11)	0.0472*	89.56 (44.16)	96.90 (47.72)	0.4576

pMax, Maximal MMT dose before enter this trial; SS, steady‐state MMT dose in this trial; Max, Maximal MMT dose during this trial; avg, average; S.D.: standard deviation; [E2]: 17β‐oestradiol; [P4]: pregn‐4‐ene‐3,20‐dione (progesterone). *P < 0.05; **P < 0.01.

We therefore would like to test whether oestrogen participates in methadone metabolism (Table 3) and MMT efficacy (Table 4) in second cohort patients. The correlation analysis found E2 levels significantly correlated with S‐form (*r* = 0.25822, *P* = 0.0146), R‐form (*r* = 0.23588, *P* = 0.0261) and total (*r* = 0.26566, *P* = 0.0119) EDDP/methadone ratio as shown in Table 3. Literature reported that R‐form methadone exerts opioid function, which related to alleviating withdrawal syndrome 41. On the contrary, S‐form is associated with the electropathological cardiac QT‐interval prolongation complications of methadone 42. In order to associate methadone metabolism to MMT therapeutic efficacy, we introduced the questionnaire 30 by which measuring the heroin using and craving after receiving MMT. The questionnaire HUC 1–6 represents the extent of mind using heroin and whether one can shift attention from heroin use, where HUC 7–14 was used to investigate the daily or weekly frequency of heroin usage, life and work disturbed, prong to anxiety emotion, desiring to use heroin and the ability to overcome heroin use 30. We scored and calculated the result in each patient to evaluate the association with methadone metabolism data. Our data showed that S‐form, R‐form and total‐EDDP/methadone ratio is significantly correlated with oestradiol (*r* = 0.25822, 0.23588, 0.26566; and *P* = 0.0146, 0.0261, 0.0119, respectively) but not associated with progesterone (Table 3). In addition, the results demonstrated that oestradiol affects both S‐form and R‐form methadone metabolism. Furthermore, the study revealed that S‐form EDDP/methadone ratio is negatively associated with medication‐related HUC 7–14 score (Table 4; *r* = −0.24246, *P* = 0.0213), but not HUC 1–6. It indicates that the higher the S‐EDDP/methadone ratio, the lower the score, and the less cravings or less dependent for heroin use. In other words, the higher the S‐form methadone metabolite, the lower is the heroin craving compulsive behaviour. Furthermore, insignificance association result of HUC 1–6 scores indicated the heroin obsessive craving, assessing the mind using of heroin is independent to methadone metabolite.

**Table 3:** Correlations between E2 and ratios of methadone metabolism

S‐ED/Met.	R‐ED/Met.	Total‐ED/Met
[E2]		
*R* = 0.25822	*R* = 0.23588	*R* = 0.26566
*P* = 0.0146	*P* = 0.0261	*P* = 0.0119

S‐ED/Met., S‐enantiomer EDDP/Methadone ratio; R‐ED/Met., R‐enantiomer EDDP/Methadone ratio; Total‐ED/Met., total‐EDDP/Methadone ratio; Patient # = 90.

**Table 4:** Correlations between HUC score and ratios of methadone metabolism

HUC1_6	HUC7_14
S‐ED/Met.	
*R* = −0.08044	*R* = −0.24246
*P* = 0.4511	*P* = 0.0213*
R‐ED/Met.	
*R* = 0.01124	*R* = −0.13545
*P* = 0.9163	*P* = 0.2031
Total‐ED/Met.	
*R* = −0.03765	*R* = −0.20329
*P* = 0.7246	*P* = 0.0546

HUC1_6 = HUC1 + HUC2 + HUC3 + HUC4 + HUC5 + HUC6. HUC7_14 = HUC7 + HUC8 + HUC9 + HUC10 + HUC11 + HUC12 + HUC13 + HUC14. Patient # = 90.

Together, Tables 1, 2, 3, 4 provide strong association that oestrogen signal might contribute to MMT sex biased dosing, alter methadone pharmacology actions, through which abate methadone efficacy in patients.

### Oestrogen mitigates methadone antinociception and rewarding behaviour in mice

**Figure 1:**
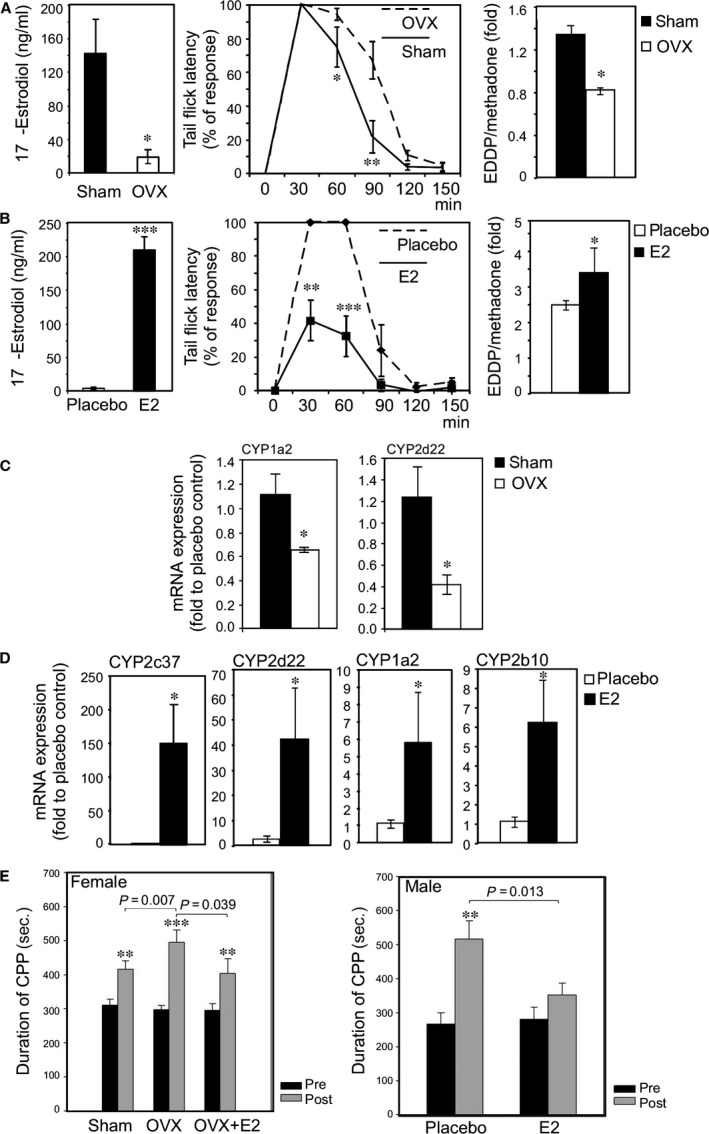
Oestrogen signal mitigated the antinociception and rewarding effects of methadone by up‐regulating the expression of CYP enzymes causing an increase in the metabolism of methadone. (**A**) Ovariectomy (OVX) reduced systemic 17β‐oestradiol levels (left figure), prolonged methadone antinociception duration (middle figure) and increased the catabolism of methadone in mice (right figure). The methadone (10 mg/kg) was injected into female mice (sham surgery *n* = 10; OVX surgery *n* = 10), tail flick latency was measured at 30, 60, 90, 120 and 150 min. after injection. OVX reduced EDDP‐to‐methadone ratio (EDDP/Methadone). The female mice were injected with methadone (10 mg/kg), and then, blood was collected 30 min. after injection to measure EDDP/methadone. (**B)** Injection of 17β‐oestradiol (E2) increased systemic 17β‐oestradiol levels (left figure), reduced the duration of methadone‐induced analgesia (middle figure) and enhanced the catabolism of methadone in mice (right figure). Briefly, the male mice were injected with methadone (10 mg/kg; vehicle‐injection, *n* = 10; E2‐injection, *n* = 10), and tail flick latency was measured at 30, 60, 90, 120 and 150 min. after injection. E2‐injection increased EDDP/methadone ratio in male mice. The male mice were injected with methadone (10 mg/kg), and blood samples were collected 30 min. after injection to measure EDDP/methadone. (**C)** OVX reduced the mRNA expression of methadone catabolic enzymes CYP2d22, CYP1a2 in female mice livers. (**D)** E2‐injection increased the mRNA expression of methadone catabolic enzymes CYP2c37, CYP2d22, CYP1a2 and CYP2b10 in male mice livers. (**E)** OVX lengthened preference duration in the Conditioned Preference Place (CPP) test. An injection to restore E2 reversed this effect in OVX mice. Briefly, three groups of female mice (sham, *n* = 8; OVX,* n* = 8; OVX + E2, *n* = 8) received 10 consecutive daily injections of methadone (10 mg/kg per day). CPP was measured at day 0 and day 10. (**F)** Injection of E2 shortened duration of preference in the CPP test in the male mice. Briefly, two groups of 16‐week‐old male mice (vehicle, *n* = 8; E2‐injection, *n* = 8) received 10 consecutive daily injections of methadone (10 mg/kg per day). CPP was measured at day 0 and day 10. *indicates a *P*‐value < 0.05, **<0.01 and ***<0.001.

We have tested E2 effect on the methadone antinociception in mice by removing endogenous female hormones (OVX), or exogenous injection of E2 in male. Figure 1A shows OVX female mice exhibited lowered 17β‐oestradiol level and prolonged methadone‐induced antinociceptive effects (tail flick test). Meanwhile, the serum EDDP/methadone ratio is also significantly decreased in the OVX mice. On the contrary, E2 supplement leads to elevated 17β‐oestradiol and ablated antinociceptive response with raised EDDP/methadone ratio in male mice (Fig. 1B). This results implicating oestrogen could influence methadone, in part, go through facilitating methadone metabolism.

In order to delineate the relation of oestrogen on methadone metabolism and CYPs expressions, the related liver enzymes mRNA expressions were measured (Fig. 1C). The liver CYPs mRNA expression (CYP1a2: an ortholog of human CYP1A2; CYP2d22: an ortholog of human CYP2D6) was significantly decreased in OVX female group as compared to sham control (Fig. 1C). On the contrary, the CYP1a2, CYP2d22 and CYP2c37 (an ortholog of human CYP2C19) and CYP2b10 (an ortholog of human CYP2B6) were increased in E2 supplement male mice (Fig. 1D).

The conditioned place preference (CPP) test was also used to test the oestrogen effect on methadone‐induced rewarding behaviour. As shown in Fig. 1E, chronic methadone injection prolonged CPP duration, while OVX further enhances this effect. Restoration of E2 in OVX mice could reverse OVX‐mediated CPP duration in female mice. On the other hand, chronic methadone administration could also prolong CPP duration in male mice, while co‐administration of E2 could diminish methadone effect (Fig. 1F).

To sum up, the result in Fig. 1 demonstrated that oestrogen could up‐regulate liver CYPs to facilitate methadone metabolism; therefore, ablate its antinociception and rewarding effect in mice.

### Differential regulation of oestrogen on ERE‐SNPs

**Figure 2:**
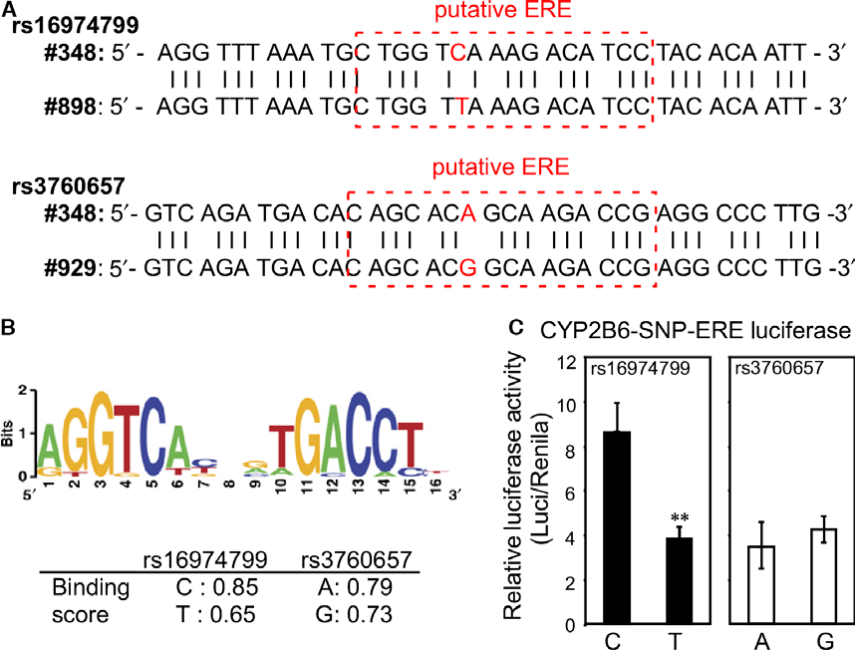
The Oestrogen‐CYP/ERE/SNP‐MMT dosing axis. (**A)** Two CYP2B6‐ERE‐SNP rs16974799 (C from patient #348 *versus* T from #898) and rs3760657 (A from patient #348 *versus* G from patient #929) sequences are shown in the upper panel. (**B)** The reference ERE sequence is shown in the upper panel. The CYP2B6‐ERE‐SNP alignment score is shown in the lower table. (**C)** The higher the ERE alignment score of CYP2B6‐ERE‐SNP, the higher the ER transactivation detected in the hepatoma HepG2 cells. The CYP2B6‐ERE‐SNP sequences were cloned, constructed into pGL3‐basic luciferase plasmid and co‐transfected into hepatoma HepG2 cells with pRenila‐Thymidine Kinase (pRL‐TK; transfection control) to measure ERE transactivation function. The relative luciferase activity was significantly higher in C‐genotype of rs16974799 CYP2B6‐ERE‐SNP compared to T‐genotype, while luciferase activities in A‐ and G‐genotype of rs3760657 CYP2B6‐ERE‐SNP were not noticeably different. **P < 0.01.

As we have illustrated E2‐CYP‐MMT dosing axis, we further examined direct regulation of E2/ER signals on CYP expression. We compared two ERE‐SNPs site, where one of them is significantly associated with Max dose of MMT (rs16974799) and the other is not (rs3760657). We calculated the putative ERE sequence alignment score by the BEST software with input of the known ERE as reference 42. (Fig. 2A). The ERE‐SNP rs16974799 (patient #348: C allele *versus* #898: T allele) C allele gained higher ERE alignment score than that of T allele (0.85 *versus* 0.65). On the other hand, the ERE‐SNP rs3760657 (patient #348: A allele *versus* #928: G allele) A and G alleles exhibited fewer contrast on alignment score (0.79 *versus* 0.73) (Fig. 2B). That implicated CC genotype of rs16974799 exerts higher oestrogen/ER transactivation function but not on rs3760657 SNP locus. Furthermore, we constructed ERE‐SNPs into pGL3‐basic luciferase reporter plasmid to test the levels of ERE‐SNP genotype ER transactivation function in hepatoma cells. The CC genotype of rs16974799 demonstrated significant higher activity than TT genotype, while comparable luciferase activity in AA and GG genotype of rs3790957 (Fig. 2C).

### Preclinical trial: Targeting oestrogen signal *via* TMX in MMT program

**Figure 3:**
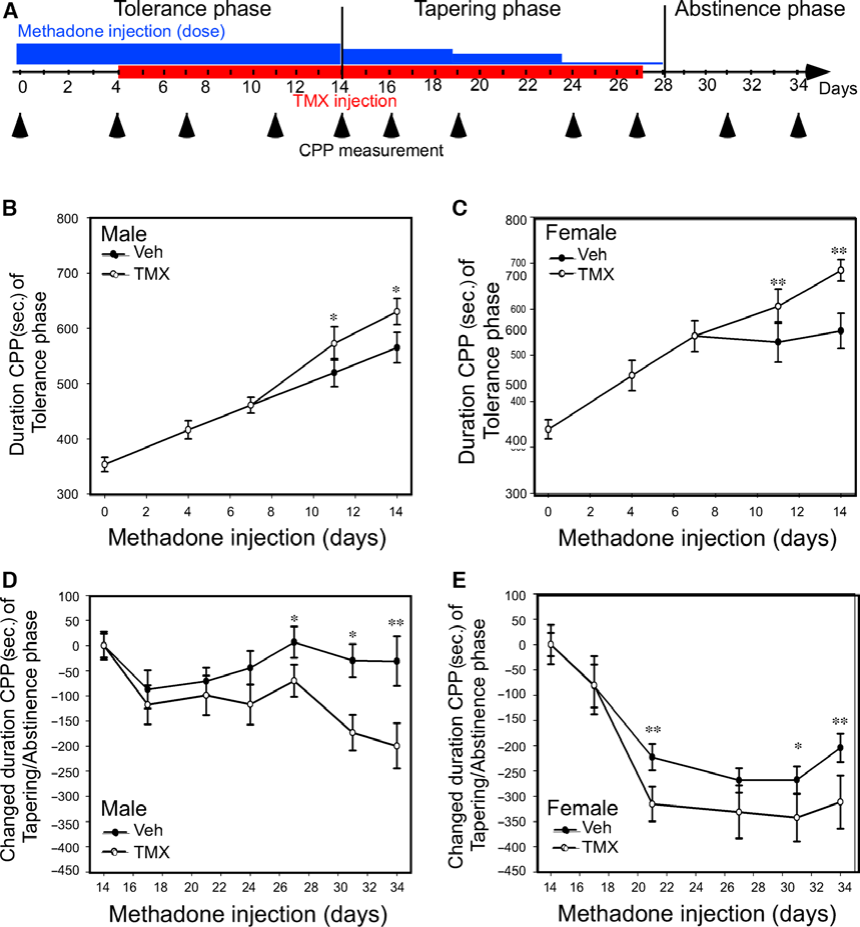
Tamoxifen (TMX; 100 ug/mice/day) injection altered the MMT tapering and abstinence phases in mice. (**A)** Experimental procedures performed during the tolerance, tapering and abstinence phases of MMT (blue coloured). In the tolerance phase, the mice were injected with 10 mg/kg methadone for 14 consecutive days, which was gradually decreased to 5 mg/kg per day for days 14~19 to 2.5 mg/kg for days 19~23 and to 1.25 mg/kg for days 23~28) in the tapering phase for another 14‐days. After 28 days, mice were injected with PBS for another eight consecutive days in abstinence phase. (**B** and **C)**. TMX increased preference duration in the tapering phase in the methadone‐treated mice receiving the CPP test. The male mice (*n* = 16; B) and female mice (*n* = 10; **C**) were enrolled into a 14‐day methadone injection program. Co‐injection with vehicle or TMX began on day 7 of the program. CPP was measured on days 0, 4, 7, 11 and 14 and showed as duration of CPP (sec.). (**D** and **E)** TMX shortened preference duration in both the male mice (**D**) and female mice (**E**) during the tapering and abstinence phases. After the tolerance phase experiment, the mice then began the tapering and abstinence phases of their MTT program, which involved the administration of gradually decreased methadone doses with either the vehicle or TMX co‐injected. The CPP duration at day 14 (end of tolerance phase) is set as tapering onset CPP time in this experiment. Changed CPP duration is defined as a result of subtracting each CPP time‐point from ground from tapering onset CPP times. *denotes a *P*‐value < 0.05, **<0.01 and ***<0.001.

While heroin/morphine addiction patients enter MMT program, it always needs to titrate methadone doses and often develop to tolerance phase and then move on to tapping and abstinence phases. Conceptually, at the beginning of methadone tolerance phase, the dosing goal is to reduce withdrawal syndrome and avoid drug‐seeking behaviour. After tolerance phase, the patient can be stabilized under MMT program and then gradually reduce doses in tapping phase. However, before the addiction of methadone is formed, social/family supporting system chip‐in to help patient gradually get rid of methadone (abstinence phase). As we have delineated the relation of E2‐CYP‐EDDP/methadone‐MMT efficacy, we would like to appeal a translational approach (Fig. 3A). We tested the hypothesis that co‐administration of Tamoxifen, TMX (one of the Selective Estrogen Receptor Modulator (SERM)) 43, in the chronic methadone injection procedure (tolerance phase) to observe TMX effect on methadone induced addiction by measuring CPP duration and then gradually reduce methadone dose (tapping and abstinence phases) to see TMX effect on CPP duration. Our data showed methadone reward response can be observed after seven‐day injection in both male (Fig. 3B) and female (Fig. 3C) mice; and co‐administration of TMX could facilitate methadone‐induced CPP duration at day 11 and day 14 (Fig. 3B, 3C). Furthermore, while we gradually reduce methadone dose in same mice and measure CPP duration reduction, we found the reduction velocity is faster in TMX co‐administration mice compared to placebo co‐administration (Fig. 3D and E).

Together, the data in Figure 3 indicating that targeting oestrogen signal *via* TMX co‐administration not only enhance rewarding response during tolerance phase, but also shorten tapping and abstinence phases of MMT program.

## Discussion

This study illustrated the roles of oestrogen signal on methadone metabolism, through which alters MMT efficacy. This report approached hypothesis directly into human with two study cohorts. The E2‐CYPs‐methadone metabolism axis was delineated by *in vivo* and *in vitro* experiments. The pathway characterization of oestrogen‐CYP‐ERE‐SNP was performed in genomic levels; and oestrogen‐methadone chiral‐chemical metabolomics was analysed to link with patient drug seeking behaviour. At last, the proof‐of‐concept preclinical trial creates a translational value for future MMT therapy. Therefore, the impact of this study might be extension of the followings.

### From modulating oestrogen signal to MMT outcome

Methadone prevents withdrawal and limits cravings 7 but additional methadone may increase cravings for heroin 44. Methadone dosing is interfering by methadone metabolism, plasma level, heroin craving and withdrawal and many other factors. High dropout of MMT and heroin relapse was related to low dose of methadone 45, and methadone dose was highly variable among countries. Our clinical data showed CYP2B6 and plasma E2 influenced methadone dosing. The measurement of E2 or CYP2B6 polymorphism might be useful for dosing adjustment for clinical methadone treatment.

The cycle of addiction is constituted with positively reinforcement of drug‐induced euphoria and negatively reinforcement of withdrawal or craving dysregulation 46. In other words, in the induction or early phase of addiction, the drug use trigger trended intention for getting euphoria (desire). Over time, chronic drug use could disrupt reward circuits and produce dysphoric states; at this time, the drug use trigger trended to avoid suffering. Our findings also indirectly approve this theory. The ratio of S‐EDDP/S‐methadone was negatively correlated with heroin compulsive use behaviour of HUC 7–14 rather than obsessive thought of questionnaire HUC 1–6. This implies that lower metabolic rate of S‐methadone may drive the drug using behaviours to prevent the body suffering, even who was not want to get drugs in his/her mind.

### Systemic *versus* neuronal action of oestrogen effect in MMT program

E2 has been reported to be involved in various neuronal activities, for example pain sensation, mood, seizures susceptibility and stroke or Alzheimer's disease neuroprotection 47. Among those human diseases‐related activities, there is an important reciprocal regulatory mechanism exists in E2 and opioid system in the CNS. For example, E2 could rapidly attenuate the ability of MOR to hyperpolarize hypothalamic (β‐endorphin) neurons 48. In reverse, opioid system also influences the prolactin release through the oestrogen regulation 49 or abolishes oestrogen‐induced antinociception and hyperalgesia 50. The sensitivity and the expressions of MOR reduced by E2 might decrease the responsiveness of MOR against its ligands (*e.g*. morphine or methadone) 48, 51, 52, 53 and consequently increase the need of elevating ligands concentration to reach the same cellular and systemic effects. In addition to MOR effect, E2 could also regulate neurotransmitters release, for example β‐endorphin, serotonin, choline and dopamine 54.

Additionally, E2 could feedback through the Hypothalamus–Pituitary Organs circuit (gonadotropin‐releasing hormone [GnRH]‐Luteinizing hormone [LH])‐E2 *per se*.) to complete this reciprocal regulation. For example, CNS E2 level might influence the endocrine circuit *via* opioid system to regulate the plasma E2 level. Quesada and Micevych (2008) showed that E2 increased the nociceptin receptor (NOPR) number (Bmax) and maximal GTPγS binding (Emax) in the cell membrane of mediobasal hypothalamus. Besides, intracerebroventricular injected or microinjected nociceptin (N/OFQ) into the hypothalamus inhibited the release of GnRH from the hypothalamus and then to decrease plasma LH levels 55.

Although E2 regulatory role in CNS opioid receptor has been reported, whether this circuit participates in MMT program is not weight yet. This study describes SERM enhances methadone effect and reduces methadone liability through its metabolism in the peripheral. It is of great interests to evaluate whether changing E2 signal could also alter neuronal activity upon methadone administration. The reason is due to a significant population of hepatic function impairment or multiple toxic agents' ingestion, *for example* alcohol or mix‐opiates exists in patients. As there are whole category of SERMs were reported to modulate cell/organ‐specific ERs activity, finding neuronal‐specific SERM might suffice such needs in clinics.

### Altering methadone metabolism to the management of heroin/morphine addiction patients

**Figure 4:**
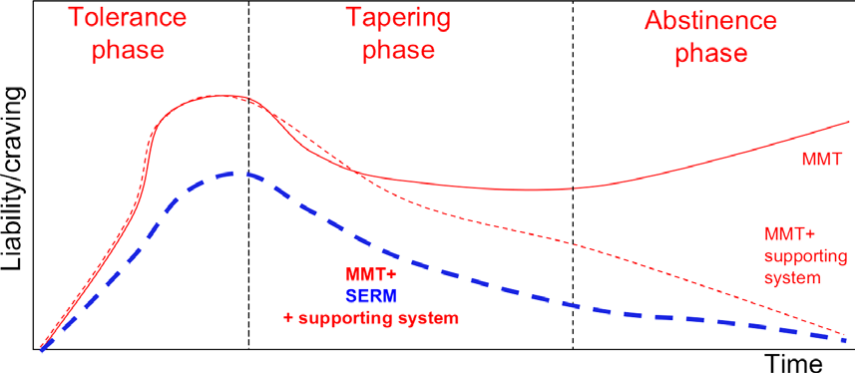
The hypothetical model illustrates changes in methadone liability and craving during MMT in three different treatment approaches, which links to three outcome scenarios. The three treatment approaches including receiving methadone alone, methadone + support system or methadone + SERM + support system during the tolerance, tapering and abstinence phases. The red‐solid line representing methadone alone group indicated methadone liability to quickly increase over the tolerance phase and decline over the taper phase, but then increase again during the abstinence phase. The red‐dashed line, representing methadone + support system group, shows methadone liability increasing at a rate comparable to that of methadone alone in the tolerance phase and then declining in tapering and abstinence phases. The blue‐dashed line representing methadone + SERM+support system shows methadone liability increasing but to a lower degree than that it did in mice receiving methadone alone in the tolerance phase. It declined to even lower degree than it did in mice receiving methadone+support in both the tapering and abstinence phases.

Balancing withdrawal syndrome alleviation and liability prevention of MMT is one important principle in managing heroin/morphine addictive patients 24. There are three phases, tolerance, tapping and abstinence, in MMT program that patient encounters as clinicians intensely monitored. In the past, most MMT receivers fall on two scenarios of therapy outcome (Fig. 4, red‐lines). First, patient takes higher methadone doses during the tolerance phase, following with a dose descending in the tapping and abstinence phases. However, liability gradually increases and switches to methadone addictive mode. Second, the patient receives regular MMT program, and add with social/family supporting system, through which to help patients gradually get rid of methadone or other opioids control. However, it really needs enormous resources/support to make it function. Our study describes the third scenario (Fig. 4; blue‐dashed line) that co‐administration of TMX (SERM) in MMT program showing increased opioid response in both sexes mice during tolerance phase. On the other hand, while methadone doses descending in the tapping and abstinence phases, TMX co‐administration also showed faster decline of CPP duration. This implicating the rewarding response is lowered in TMX co‐administration mice.

The significance of the translational study is that the higher dosing or difficult treating MMT patients might use SERM as combination therapy. Furthermore, co‐administration of SERM in MMT program provides self‐driven defence on methadone liability.

## Conclusion

This study illustrates a novel strategy on handling MMT receivers with less dosing, quick extinction of opioid replacement therapy for heroin/morphine addiction patients. The examination of ERE‐SNP in patients could serve as precision medication markers, and the clinical trial using SERM (TMX, *per se*) in MMT regimen is also suggested.

## Conflict of interest

The Taiwan and US patent ‘Pharmaceutical combination for treating withdrawal syndromes and use thereof’ has been filed, and WL Ma, YC Chiang, RY Wang, CL Huang are co‐inventers. Other authors stated no interest conflict.

## Supporting information


**Table S1** Characteristics of two cohorts study subjects on MMT.
**Table S2** Primer list for SNP and qRT‐PCR.
**Table S3** The association of ERE‐SNPs in MMT Max.
