# Supplementary material for: Reduced dosing and liability in methadone maintenance treatment by targeting oestrogen signal for morphine addiction
Source: J Cell Mol Med. 2017 Jul 12;21(12):3552–64. doi: 10.1111/jcmm.13266 (PMC5706516; doi:10.1111/jcmm.13266)
Supplement: Supplementary file 1 — Table S1 Characteristics of two cohorts study subjects on MMT. Table S2 Primer list for SNP and qRT‐PCR. Table S3 The association of ERE‐SNPs in MMT Max. [file JCMM-21-3552-s001.pdf]

Supplement Table 1. Characteristics of two cohorts study subjects on MMT.

	cohort-1 N=93	cohort-2 N=17	cohort-1 N=150	cohort-2 N=35	cohort-1 N=78	cohort-2 N=38
	maxdose≦50; Mean±SE		50<maxdose<100;Mean±SE		maxdose≧100;Mean±SE	
Gender						
male	71	13	124	30	59	23
female	22	4	26	5	19	15
Age (y)	42.08±0.75	42.59±1.60	43.05±0.61	41.43±1.10	40.21±0.81	37.47±1.20
BMI (Kg/M^2)	22.83±0.34	24.11±0.94	22.81±0.24	24.05±0.69	22.35±0.28	23.57±0.77
Maxdose(mg/day)	38.33±1.13	41.18±2.25	75.10±0.88	74.29±1.94	115.06±1.73	133.29±6.30
SGOT	40.80±4.28	38.56±5.25	43.00±2.39	43.64±3.45	46.25±6.71	47.52±4.95
SGPT	48.14±4.78	29.1±4.83	55.41±4.32	28.91±3.05	56.53±8.43	39.96±6.04
r-GT	46.12±6.83	57.42±32.33	37.03±2.36	35.61±6.07	37.25±5.81	58.37±12.59
HIV_Ab (-/+)	91/0	9/1	145/5	21/3	76/2	24/2

Supplemental Table 2, Primer list for SNP and qRT-PCR

Primers for SNP genotyping

Gene (SNP_ID)	1st-PCR Primer	2nd-PCR Primer
OPRk-1 (rs6473799)	5'-ACGTTGGATGCAGGGAAAATGCAGTCCTTC	5'-ACGTTGGATGAGAGCTCCCAAACTCATCTC
OPRL-1 (rs7271530)	5'-ACGTTGGATGCCTGTTAGAGAGGGATTATG	5'-ACGTTGGATGGAGCTTGGCACAGGTATTTG
OPRL-1 (rs6010717)	5'-ACGTTGGATGACGTGGATGGCACCATCTTC	5'-ACGTTGGATGGCGAGCCTTCGGAGAATTAT
OPRD1 (rs2236860)	5'-ACGTTGGATGTGGTATGAATCCCTGCTCTG	5'-ACGTTGGATGGAAACTGAGGCTCAAAGAGG
OPRL (rs2229205)	5'-ACGTTGGATGACGTCCGCACGTCCAGCAAA	5'-ACGTTGGATGAACGGGAACACCGACAACAG
CYP2C19 (rs11528090)	5'-ACGTTGGATGTGGACGGTTTGTGTTGAAGG	5'-ACGTTGGATGCTCAGTCTACCTCCCTATCC
CYP2C19 (rs6583954)	5'-ACGTTGGATGTTAGGAGTACTGCTGTGGAG	5'-ACGTTGGATGTGGAATCCACCTAGAACTCC
CYP2B6 (rs16974799)	5'-ACGTTGGATGGGTTCAGCGAATTGTGTAGG	5'-ACGTTGGATGCATCTGCAATGTGAGGAGTG
CYP2B6 (rs3760657)	5'-ACGTTGGATGCAGCATGGACTTTCCTGAAC	5'-ACGTTGGATGGAGCACCCAATCTTAGTGTC
CYP1A2 (rs4646425)	5'-ACGTTGGATGCTTCCTCCCAATAACACCAG	5'-ACGTTGGATGTCTGGTGTCACGTTGCTTCC

Primers for RT-PCR

Gene name	Forward Primer	Reverse Primer
CYP1a2	5’-GGCCACTTCGAACCAGTCAGCC	5’-ATCTCCTCGCTCTTCCGGGGG
CYP2b10	5’-CTTGGCCCGCTGATTGGGCTG	5’-TGCCTTGGAGCCCTGGAGATTT
CYP2c37	5’-TCCTGGGCTGTGCTCCTTGC	5’-TGCAAATCTGCAACCAAGGGCTG
CYp2d22	5’-TCAGAACGCACCGGTAGCTGGA	5’-CCGGTCGGCAGTCTCATGGC

Supplmental Table 3, The association of ERE-SNPs in MMT Max

ERE-SNP site	Genotypes	≦50 (n=79)	51-99 (n=127)	≧100 (n=65)	p-value
OPRk-1 (rs6473799)	AA	68(30.0)	103(45.4)	56(24.7)	0.4472
	GG	10(23.3)	24(55.8)	9(20.9)	
OPRL-1 (rs7271530)	TT	22(24.7)	47(52.8)	20(22.5)	0.0492
	CC	14(33.3)	12(28.6)	16(38.1)	
	CT	43(31.4)	67(48.9)	27(19.7)	
OPRL-1 (rs6010717)	GG	30(25.2)	61(51.3)	28(23.5)	0.1278
	CC	12(50.0)	6(25.0)	6(25.0)	
	CG	36(29.3)	59(48.0)	28(22.8)	
OPRD-1 (rs2236860)	GG	61(29.8)	94(45.9)	50(24.4)	0.6183
	AA	1(20.0)	4(80.0)	0(0)	
	AG	17(28.3)	29(48.3)	14(23.3)	
OPRL (rs2229205)	CC	60(30.5)	95(48.2)	42(21.3)	0.1649
	TT	0(0)	3(42.9)	4(57.1)	
	CT	19(28.8)	29(43.9)	18(27.3)	
CYP2C19 (rs11529090)	TT	33(25.4)	62(47.7)	35(26.9)	0.5222
	GG	14(37.8)	15(40.5)	8(21.6)	
	GT	29(30.9)	46(48.9)	19(20.2)	
CYP2C19 (rs6583954)	CC	34(29.6)	53(46.1)	28(24.4)	0.997
	TT	9(30.0)	14(46.7)	7(23.3)	
	CT	36(29.0)	60(48.4)	28(22.6)	
**CYP2B6 (rs16974799)**	**CC**	**41(23.8)**	**83(48.3)**	**48(27.9)**	**0.0441** *
	**TT**	**5(55.6)**	**3(33.3)**	**1(11.1)**	
	**CT**	**33(37.1)**	**41(46.1)**	**15(16.9)**	
CYP2B6 (rs3760657)	AA	61(30.1)	86(42.4)	56(27.6)	0.0909
	GG	2(2.6)	6(4.0)	1(2.4)	
	AG	15(26.3)	34(59.7)	8(14.0)	
CYP1A2 (rs4646425)	CC	69(29.7)	109(47.0)	54(23.3)	0.8728
	TT	2(40.0)	2(40.0)	1(20.0)	
	CT	7(21.9)	16(50)	9(28.1)	
